# The *Vibrio* type VI secretion system induces intestinal macrophage redistribution and enhanced intestinal motility

**DOI:** 10.1128/mbio.02419-24

**Published:** 2024-11-22

**Authors:** Julia S. Ngo, Piyush Amitabh, Jonah G. Sokoloff, Calvin Trinh, Travis J. Wiles, Karen Guillemin, Raghuveer Parthasarathy

**Affiliations:** 1 Institute of Molecular Biology, University of Oregon, Eugene, Oregon, USA; 2 Department of Physics, University of Oregon, Eugene, Oregon, USA; 3 Department of Molecular Biology & Biochemistry, University of California, Irvine, California, USA; 4 Humans and the Microbiome Program, CIFAR, Toronto, Ontario, Canada; University of Hawaii at Manoa, Honolulu, Hawaii, USA

**Keywords:** *Vibrio*, zebrafish, macrophages, type VI secretion system, peristalsis

## Abstract

Intestinal microbes, whether resident or transient, influence the physiology of their hosts, altering both the chemical and the physical characteristics of the gut. An example of the latter is the human pathogen *Vibrio cholerae’s* ability to induce strong mechanical contractions, discovered in zebrafish. The underlying mechanism has remained unknown, but the phenomenon requires the actin crosslinking domain (ACD) of *Vibrio*’s type VI secretion system (T6SS), a multicomponent protein syringe that pierces adjacent cells and delivers toxins. By using a zebrafish-native *Vibrio* and imaging-based assays of host intestinal mechanics and immune responses, we find evidence that macrophages mediate the connection between the T6SS ACD and intestinal activity. Inoculation with *Vibrio* gives rise to strong, ACD-dependent, gut contractions whose magnitude resembles those resulting from genetic depletion of macrophages. *Vibrio* also induces tissue damage and macrophage activation, both ACD-dependent, recruiting macrophages to the site of tissue damage and away from their unperturbed positions near enteric neurons that line the midgut and regulate intestinal motility. Given known crosstalk between macrophages and enteric neurons, our observations suggest that macrophage redistribution forms a key link between *Vibrio* activity and intestinal motility. In addition to illuminating host-directed actions of the widespread T6SS protein apparatus, our findings highlight how localized bacteria-induced injury can reshape neuro-immune cellular dynamics to impact whole-organ physiology.

**IMPORTANCE:**

Gut microbes, whether beneficial, harmful, or neutral, can have dramatic effects on host activities. The human pathogen *Vibrio cholerae* can induce strong intestinal contractions, though how this is achieved has remained a mystery. Using a zebrafish-native *Vibrio* and live imaging of larval fish, we find evidence that immune cells mediate the connection between bacteria and host mechanics. A piece of *Vibrio*’s type VI secretion system, a syringe-like apparatus that stabs cellular targets, induces localized tissue damage, activating macrophages and drawing them from their normal residence near neurons, whose stimulation of gut contractions they dampen, to the damage site. Our observations reveal a mechanism in which cellular rearrangements, rather than bespoke biochemical signaling, drives a dynamic neuro-immune response to bacterial activity.

## INTRODUCTION

Intestinal microorganisms, whether commensal or pathogenic, impact their hosts through a wide range of processes (1–6). Though chemical transformations related, for example, to nutrient metabolism or neurotransmitter synthesis are intensely studied, transformations of the physical environment remain underexplored, likely due to the difficulty of characterizing the gut environment *in situ*. In earlier work (7), we showed that the human pathogen *Vibrio cholerae* (El Tor strain C6706) induces strong mechanical contractions in the intestines of zebrafish, a consequence of the bacterial type VI secretion system (T6SS), a syringe-like apparatus with which *V. cholerae*, like nearly 25% of all Gram-negative bacteria, can inject effector proteins into adjacent prokaryotic or eukaryotic cells. Enhanced gut contractions, moreover, required the actin crosslinking domain (ACD) present at the C-terminus of the *Vibrio* T6SS spike protein VgrG-1, indicating a eukaryotic target. The nature of this connection between bacterial activity and host mechanical response has, however, remained mysterious.

To map the connection between the T6SS and host intestinal activity, we examined host responses to a *Vibrio* species isolated from and commonly found in zebrafish intestines, strain ZWU0020, which unlike the human-derived *V. cholerae* El Tor C6706, does not encode cholera toxin or toxin-coregulated pili (8). Cholera toxin can itself induce epithelial barrier damage and diarrhea (9–11). In addition to facilitating a focus on the T6SS, *Vibrio* ZWU0020, hereafter referred to simply as “*Vibrio*” for brevity, colonizes the larval zebrafish gut to about 10 times the abundance of *V. cholerae* El Tor C6706 (7), forming dense communities of planktonic, highly motile cells, shown in prior work to be capable of invading existing gut bacterial populations (12) and causing intestinal inflammation in the form of gut-localized neutrophils (13–15) and macrophages expressing the cytokine TNFα (16). Though the majority of motile *Vibrio* are found in the anterior bulb of larval zebrafish, the bacteria are present throughout the intestine, including the narrow posterior end (16). The pro-inflammatory character of *Vibrio* contributes to its characterization as a “pathobiont,” a member of the normal microbiota with a potential for pathogenicity (14–17). We studied commensal *Vibrio* colonizing larval zebrafish, a model whose transparency enables high resolution imaging of the entire intestine including visualization of epithelial tissue and labeled cells, such as immune cells and enteric neurons, in living animals (7, 16, 18–20).

We hypothesized that gut-associated macrophages were a likely intermediary between bacteria and other host cell types because of the above-mentioned observations of *Vibrio*-induced inflammation and because studies using mice and zebrafish have established communication pathways between macrophages and the enteric neurons that regulate muscular activity along the intestine. In mice, Muller et al. showed that macrophage secretion of bone morphogenic protein 2 (BMP2) activates enteric neurons, leads to hyperactive contractions in intestinal segments *ex vivo*, and increases colonic transit time (21). In zebrafish, Graves et al. found intestinal macrophages in close proximity to enteric neurons and showed that genetic ablation of the *irf8* gene, previously shown to be necessary for macrophage differentiation (22), leads to shorter transit times in larvae, though not in adults (23). How bacterial activity, especially mediated by the T6SS, might intersect with macrophage activity or mortality has remained unclear.

The imaging-based assays described here, performed in transgenic animals that allow manipulation and visualization of macrophages, revealed host tissue damage, inflammation, and changes in gut contraction dynamics induced by the *Vibrio* T6SS ACD. Strikingly, we found an ACD-dependent spatial reorganization of macrophages that appears to drive strong gut contractions while leaving the frequency of contractions unchanged. These findings not only provide insights into the ways by which resident microbes influence their hosts but also indicate an unexpected and potentially general mechanism for the regulation of intestinal mechanics via physical reorganization of relevant cell types.

## RESULTS

### The *Vibrio* T6SS actin crosslinking domain is required for enhanced intestinal contractions

To determine the role of the bacterial T6SS, specifically the eukaryote-targeting ACD, we generated a mutant of the zebrafish-native *Vibrio* with an in-frame deletion of the ACD of the T6SS spike protein VgrG-1 (Materials and Methods); we will refer to this strain as *Vibrio*^∆ACD^. We also generated a mutant strain lacking the entire T6SS gene cluster, denoted *Vibrio*^∆T6SS^ (Materials and Methods). The wild-type *Vibrio* and both mutants show identical *in vitro* growth rates in lysogeny broth (Fig. S1).

We confirmed that deletion of the ACD does not interfere with the functionality of the type VI apparatus, other than through effects directly mediated by the ACD itself, by checking that *Vibrio*^∆ACD^, but not *Vibrio*^∆T6SS^, is capable of killing bacterial targets. We performed an *in vitro* assay in which one of the *Vibrio* strains was spotted on agar plates along with another zebrafish-native species, the previously examined *Aeromonas* ZOR0001 (7, 12) (Materials and Methods). When co-spotted with the completely T6SS-deficient *Vibrio*^∆T6SS^, *Aeromonas* ZOR0001 is present at high abundances that increase monotonically with the concentration of the initial suspension, as when spotted alone, consistent with a lack of inter-bacterial killing (Fig. S2). In contrast, *Aeromonas* ZOR0001 co-spotted with *Vibrio*^∆ACD^ shows consistently low abundances that drop to nearly zero at high initial concentrations, as when spotted with wild-type *Vibrio*, consistent with inter-bacterial killing (Fig. S2).

*In vivo*, both the wild-type *Vibrio* and *Vibrio*^∆ACD^, when inoculated in mono-association with initially germ-free (GF) larval zebrafish, colonized the gut to approximately the same abundance, with the mean ± standard deviation of log_10_(bacteria per gut) being 5.0 ± 0.4 and 5.2 ± 0.4 for wild-type *Vibrio* and *Vibrio*^∆ACD^, respectively (Fig. S3).

**Fig 1:**
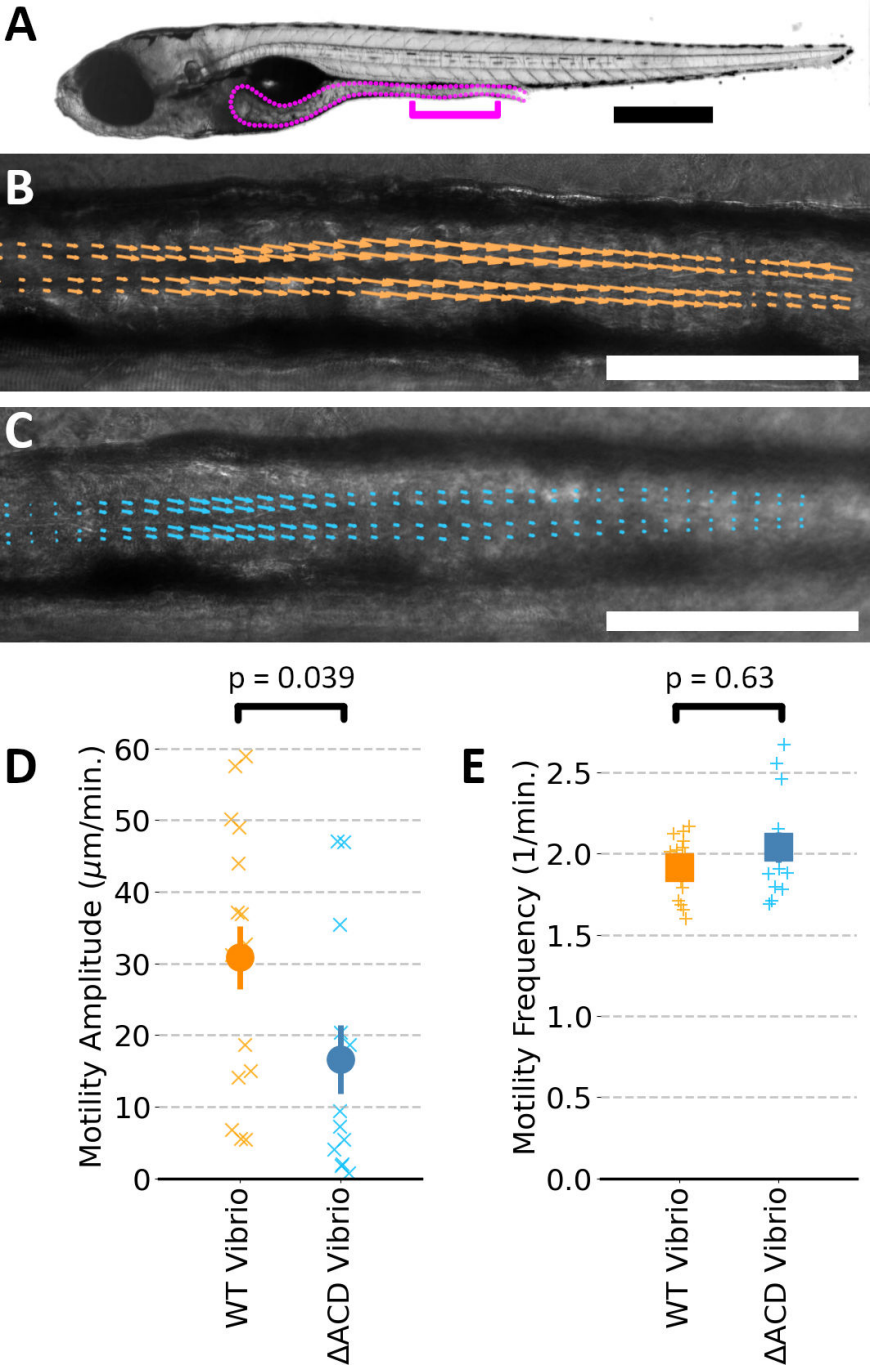
(**A**) Brightfield image of a larval zebrafish at 6 dpf, with the gut outlined. The bracket indicates the region imaged for intestinal motility assessment. Bar: 500 µm. (**B and C**) DIC images of a 6 dpf zebrafish, initially germ-free and inoculated at 5 dpf with (**B**) wild-type *Vibrio* and (**C**) *Vibrio*^∆ACD^ with superimposed arrows from image velocimetry analysis. In both images, the magnitude of arrows is assigned using the same velocity scale, the selected zebrafish are near the median motility amplitude for their condition, and the selected timepoint is near the peak of an intestinal contraction. Bar: 100 µm. See also Movies S1 to S4. (**D**) Gut motility amplitudes of 6 dpf zebrafish. The amplitude of periodic intestinal contractions is nearly 100% larger (ratio 1.86 ± 0.60) for fish mono-associated with wild-type *Vibrio* (*N* = 16) compared to *Vibrio*^∆ACD^ (*N* = 12) (*P* = 0.039). (**E**) The mean frequency of intestinal contractions is very similar between the groups (mean ± s.e.m. =1.92 ± .04 1/min. for wild-type *Vibrio*, 2.04 ± 0.09 for *Vibrio*^∆ACD^; *P* = 0.63). In panels **D** and **E**, “x”s indicate measurements of individual zebrafish; solid symbols and error bars indicate the mean and standard error of the mean, respectively.

Larval zebrafish, like other animals, exhibit periodic intestinal contractions that propagate along the length of the gut. As in past work, imaging using differential interference contrast (DIC) microscopy and analysis based on image velocimetry reveal and quantify the frequency and strength of these contractions (7, 20). GF fish were inoculated with either *Vibrio* or *Vibrio*^∆ACD^ at 5 days post-fertilization (dpf). The next day, at 24–30 h post-inoculation (hpi), we used DIC microscopy to acquire movies of 5-min duration at 5 frames per second spanning a roughly 400 µm segment of the posterior intestine of live fish (7, 20), indicated in Fig. 1A. It is readily visually apparent that contractions are stronger in fish inoculated with wild-type *Vibrio* compared to *Vibrio*^∆ACD^ (Movies S1 to S4). To quantify intestinal mechanics, we performed image velocimetry to determine the velocity field at a grid of points spanning the images (Fig. 1B and C). The cross-correlation of the velocity field shows periodic, propagating waves with a frequency revealed by Fourier analysis. The amplitude of the image velocity at the dominant frequency provides a measure of the strength of the contractions, essentially the amplitude of tissue motion corresponding to rhythmic oscillations (20). Zebrafish mono-associated with wild-type *Vibrio* showed, on average, roughly 100% larger contraction amplitude as those mono-associated with *Vibrio*^∆ACD^ (Fig. 1D; ratio 1.86 ± 0.60, *P* = 0.039), similar to effects seen previously with human-derived *V. cholerae* El Tor C6706 (7). Notably, the frequency of contractions was very similar for the two bacterial conditions (Fig. 1E), being 1.92 ± 0.04 1/min. (mean ± s.e.m.) for wild-type *Vibrio* and 2.04 ± 0.09 1/min. for *Vibrio*^∆ACD^ (*P* = 0.63).

### Macrophages downregulate gut motility and are necessary for ACD-dependent enhancement of gut contractions

**Fig 2:**
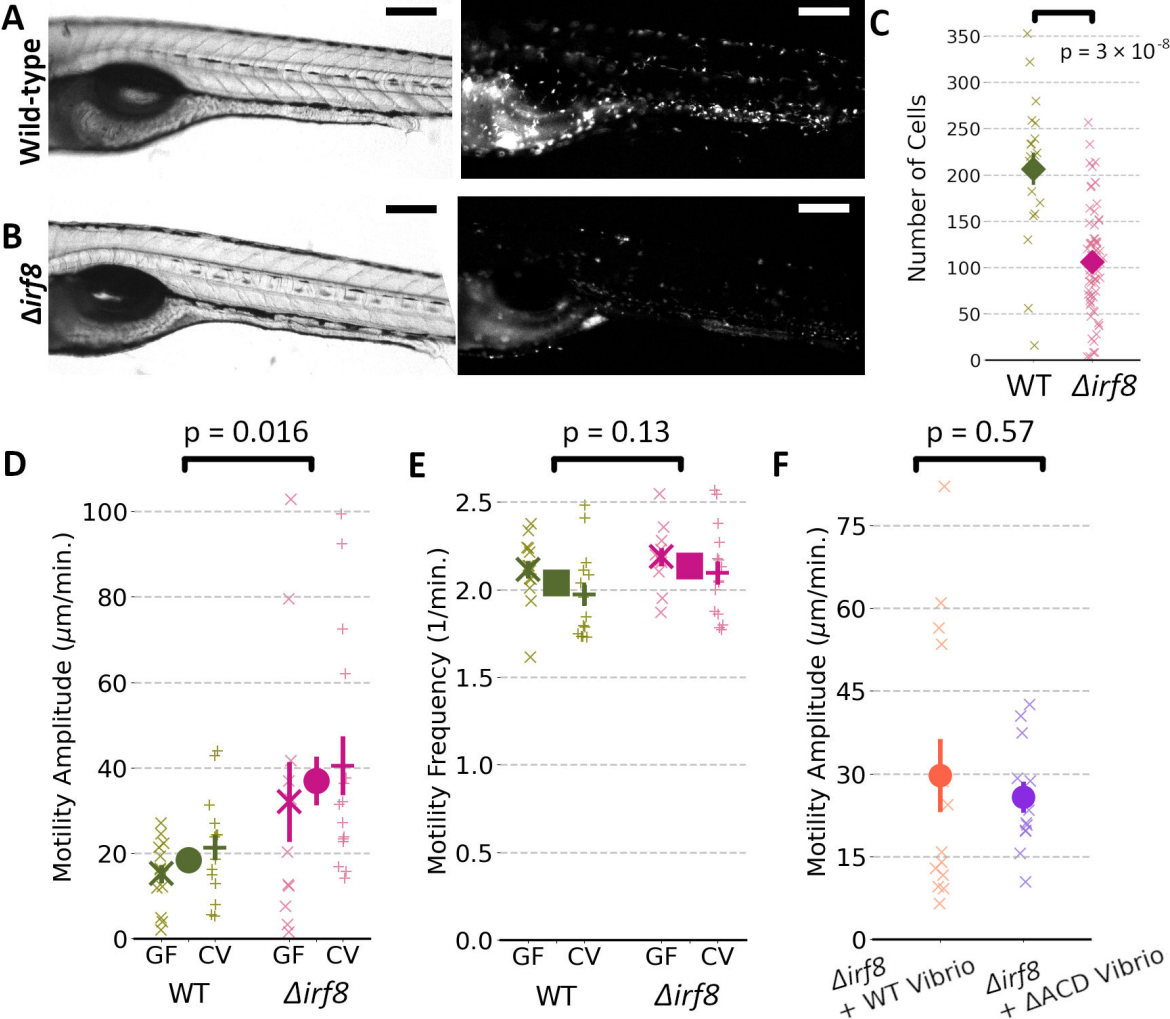
(**A**) Brightfield (left) and fluorescence (right) images of the mid-section of a 6 dpf (*Tg(mpeg1:mCherry*)) zebrafish with fluorescent macrophages. Bar = 200 µm. (**B**) Brightfield and fluorescence images from a 6 dpf ∆*irf8* zebrafish. The fluorescence image is shown at the same intensity scale as in (**A**). Bar = 200 µm. (**C**) Quantification of macrophage abundance by number of fluorescent cells in otherwise wild-type (*N* = 20) and ∆*irf8* zebrafish (*N* = 67), the latter with, on average, roughly half the number of macrophages (*P* = 3 × 10^−8^). Each “x” represents a measurement from an individual zebrafish over the mid-section as in (**A and B**). Each large symbol represents the mean, with error bars indicating the standard error of the mean (s.e.m.). The fish shown in (**A and B**) were selected for proximity of their macrophage counts to the median value of the population. (**D**) Gut motility amplitudes of 6 dpf wild-type and ∆*irf8* zebrafish. Each small “x” or “+” represents a measurement from an individual germ-free (GF) or conventionally reared (CV) zebrafish, respectively, with the large “x” or “+” indicating the mean ± s.e.m. The central circles and error bars for each fish type denote the mean and s.e.m. of the pooled data from germ-free and conventional fish, which show the amplitude of periodic intestinal contractions to be 100% larger (ratio 2.00 ± 0.37) for fish with depleted macrophages (∆*irf8*) (*N* = 26) compared to wild-type fish (*N* = 28) (*P* = 0.016). (**E**) The mean frequency of intestinal contractions is approximately equal for the two groups (mean ± s.e.m. = 2.04 ± .04 for wild-type zebrafish, 2.13 ± 0.04 for ∆*irf8* zebrafish, pooled as in (**D**); *P* = 0.13). (**F**) Gut motility amplitudes of initially germ-free 6 dpf ∆*irf8* zebrafish mono-associated with wild-type *Vibrio* (*N* = 13) or *Vibrio*^∆ACD^ (*N* = 12) are similar to each other (ratio 1.15 ± 0.29; *P* = 0.57). As in previous panels, “x”s indicate measurements of individual zebrafish; solid symbols and error bars indicate the mean and standard error of the mean, respectively.

We suspected macrophages as an intermediary between *Vibrio* activity and host intestinal motility. To quantitatively characterize the impact of macrophages on propagating intestinal contractions, we generated zebrafish with depleted numbers of macrophages. In brief, we used CRISPR-Cas9 to target the *irf8* gene, crucial for the differentiation of macrophages (23), injecting embryos at the single-cell stage with the Cas9 enzyme and the appropriate single guide RNAs to yield F0 “crispants” (24), which we will denote ∆*irf8* (Materials and Methods). Use of a transgenic zebrafish line with fluorescently labeled macrophages (*Tg(mpeg1:mCherry*)) allows quantification of macrophage number. We find that the ∆*irf8* zebrafish at 6 dpf have considerably fewer, though nonzero, numbers of macrophages, roughly half as many as otherwise wild-type fish (Fig. 2A through C). We note that the depletion of macrophages is not total.

It has been shown that larval zebrafish lacking the *irf8* gene have decreased intestinal transit time for solid food compared to wild-type siblings (23). However, the relationship between transit time and intestinal mechanics is not straightforward, as it may depend on the frequency of contractions, their strength, their coherence, and other factors. We imaged and analyzed intestinal contractions in 6 dpf wild-type or ∆*irf8* fish. Whether germ-free or conventionally reared, the macrophage-depleted ∆*irf8* fish showed highly variable contraction amplitudes with a mean roughly two times as large as that of wild-type fish (Fig. 2D). The ratio of amplitudes of ∆*irf8*/wild-type fish is 2.1 ± 0.7 and 1.9 ± 0.4 for GF and conventional (CV) fish, respectively (Fig. 2D). Note that neither the GF nor CV fish have been inoculated by *Vibrio*, suggesting, as expected, that typical multi-species consortia colonizing the zebrafish gut do not induce strong mechanical responses. Pooling the GF and CV data, the ratio of gut contraction amplitudes of ∆*irf8*/wild-type fish overall is 2.00 ± 0.37; *P* = 0.016 (Fig. 2D). In contrast to the amplitude, the frequency of contractions was nearly identical for the two types of fish (Fig. 2E), being 2.04 ± 0.04 1/min. (mean ± s.e.m.) for wild-type fish and 2.13 ± 0.04 1/min. for ∆*irf8* fish (pooled GF and CV; *P* = 0.13), matching the above-mentioned values from wild-type fish mono-associated with *Vibrio*.

To test whether a normal macrophage abundance is necessary for ACD-mediated enhancement of intestinal motility, ∆*irf8* fish were derived germ-free and then mono-associated with either wild-type *Vibrio* or *Vibrio*^∆ACD^. As above, intestinal dynamics were assessed the day after inoculation. Contraction amplitudes were similar for both sets (Fig. 2F; ratio 1.2 ± 0.4; *P* = 0.58) and similar to the value measured for ∆*irf8* fish in the absence of *Vibrio*, implying that macrophages and the *Vibrio* ACD are part of the same pathway for increasing intestinal contraction strength. Again, contraction frequencies were independent of condition (2.05 ± 0.08 1/min. (mean ± s.e.m.) for wild-type fish and 1.98 ± 0.04 1/min. for ∆*irf8* fish).

### The *Vibrio* T6SS actin crosslinking domain is required for macrophage activation

**Fig 3:**
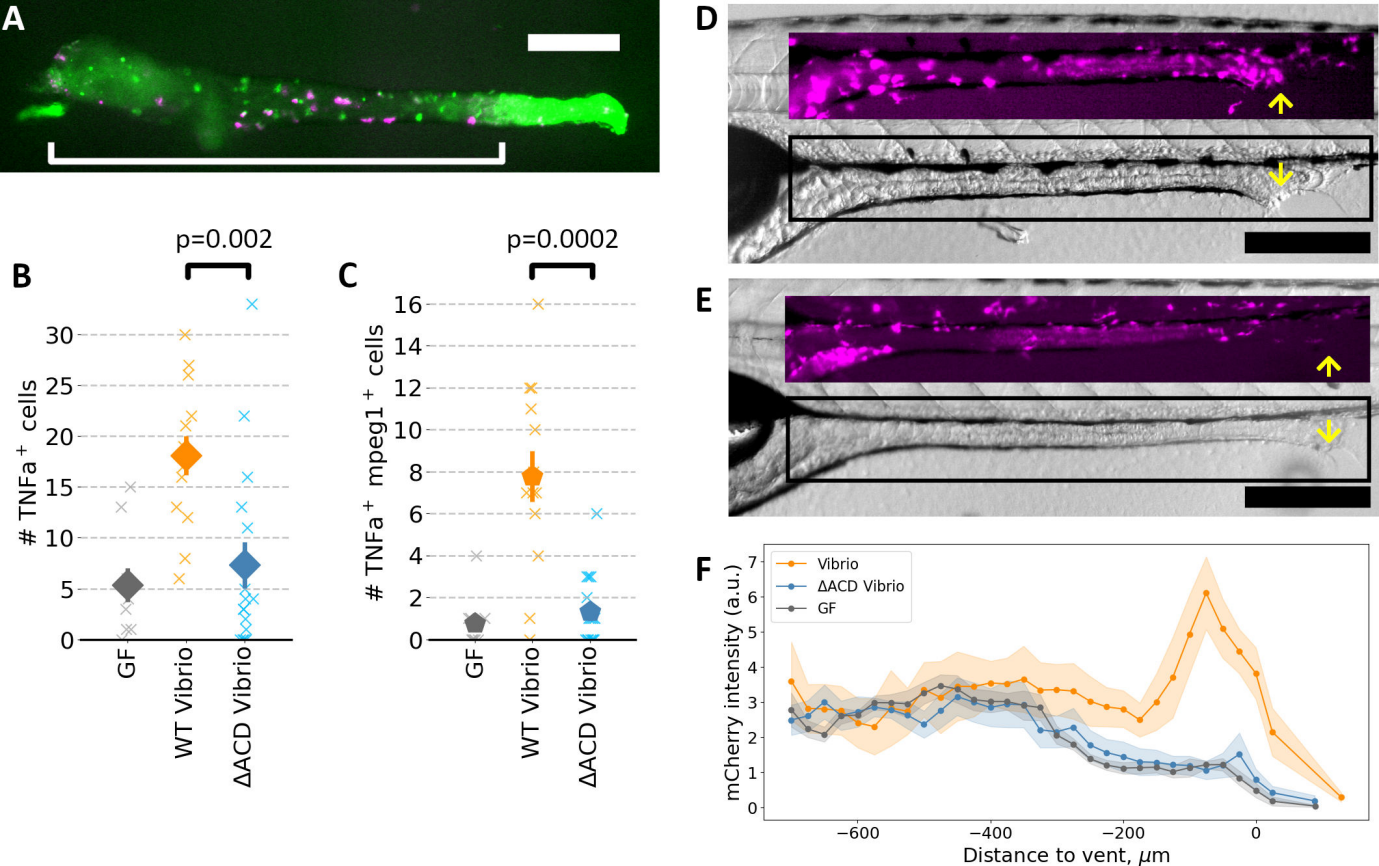
(**A**) Representative image of a dissected gut from a 6 dpf *Tg(tnfa:GFP);Tg(mpeg1:mCherry*) zebrafish mono-associated with wild-type *Vibrio*, showing gut-associated macrophages (mCherry^+^) and cells expressing the *tnfa* reporter (GFP^+^). The bracket indicates the region assessed for cell counts. Bar: 250 µm. (**B**) Numbers of gut-associated *tnfa*-positive cells in germ-free fish, fish mono-associated with wild-type *Vibrio*, and fish mono-associated with *Vibrio*^∆ACD^. The mean ± s.e.m. of GFP^+^ cells was 18.1 ± 1.9 for fish with wild-type *Vibrio* (*N* = 13), 7.3 ± 2.3 for fish with wild-type *Vibrio*^∆ACD^ (*N* = 16), and 5.3 ± 1.7 for germ-free fish (*N* = 9); comparing wild-type *Vibrio* and *Vibrio*^∆ACD^ gives *P* = 0.002. (**C**) Numbers of gut-associated *tnfa*-positive macrophages (*tnfa:GFP*^+^ and *mpeg1:mCherry*^+^). The mean ± s.e.m. of GFP^+^ and mCherry^+^ cells was 7.8 ± 1.2 for fish with wild-type *Vibrio* (*N* = 13), 1.3 ± 0.4 for fish with wild-type *Vibrio*^∆ACD^ (*N* = 16), and 1.2 ± 0.4 for germ-free fish (*N* = 9); comparing wild-type *Vibrio* and *Vibrio*^∆ACD^ gives *P* = 0.0002. In panels **B** and **C**, “x”s indicate measurements of individual zebrafish; solid symbols and error bars indicate the mean and standard error of the mean, respectively. (**D and E**) Representative brightfield and macrophage-fluorescence (insets) images of 6 dpf *Tg(tnfa:GFP);Tg(mpeg1:mCherry*) zebrafish mono-associated with (**D**) wild-type *Vibrio* and (**E**) *Vibrio*^∆ACD^. The rectangle indicates the inset region. Yellow arrows indicate the vent. Bar: 250 µm. (**F**) Mean mCherry intensity averaged over the gut as a function of anterior-posterior position, relative to the vent, for germ-free (GF) zebrafish (*N* = 6), zebrafish mono-associated with wild-type *Vibrio* (*N* = 6), and zebrafish mono-associated with *Vibrio*^∆ACD^ (*N* = 8). Shaded bands indicate the standard error of the mean at each position.

Prior work demonstrated that *Vibrio* stimulates the innate immune system as reported by the production of the pro-inflammatory cytokine TNFα (16). To clarify the role of the T6SS ACD, we derived germ-free transgenic zebrafish with fluorescent reporters of macrophages and TNFα expression (*Tg(tnfα:GFP);Tg(mpeg1:mCherry*)), inoculated the fish with bacteria, and dissected their intestines at 24 hpi to focus on gut-associated responses (Fig. 3A). Note that the *tnf*α:*GFP* fluorescence does not directly report the level of TNFα cytokines produced, but rather the expression of the gene. In addition to broad epithelial *tnf*α:*GFP* fluorescence, discrete GFP-positive cells were evident (Fig. 3A), the number of which was nearly three times greater in fish inoculated with wild-type *Vibrio* compared to *Vibrio*^∆ACD^ or to germ-free fish (Fig. 3B). TNFα is known to be produced by many cell types and to play roles in many signaling processes. In macrophages, its expression indicates an activated state. We therefore quantified the number of GFP-positive and mCherry-positive cells, that is, macrophages expressing *tnf*α, which we found to be six times greater in fish inoculated with wild-type *Vibrio* compared to *Vibrio*^∆ACD^ or to germ-free fish (Fig. 3C). The ACD, therefore, is crucial for macrophage activation.

We also noticed through simple epifluorescence imaging high mCherry (macrophage) intensity near the zebrafish posterior vent (the posterior opening of the gut) for fish mono-associated with wild-type *Vibrio* and not for other conditions (Fig. 3D through F), motivating closer examination of tissue phenotypes and cellular organization in this region.

### *Vibrio* causes posterior tissue damage but does not preferentially kill macrophages

**Fig 4:**
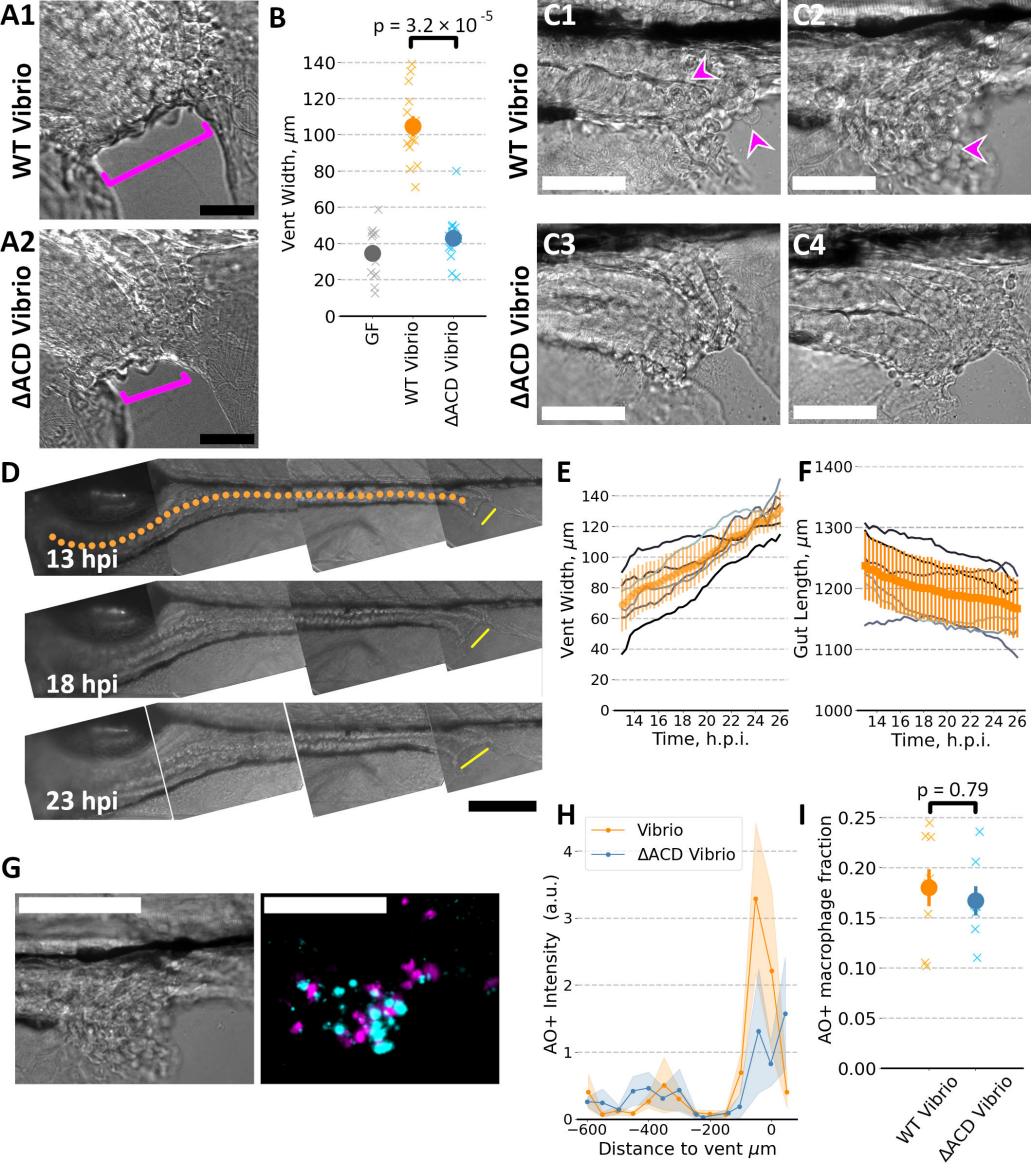
(**A**) Representative brightfield images of the vent region of 6 dpf zebrafish mono-associated with wild-type *Vibrio* or with ∆ACD-*Vibrio*, both at 24 hpi. Bar: 50 µm. (**B**) Vent width of 6 dpf zebrafish, germ-free (*N* = 13) or mono-associated with wild-type *Vibrio* (*N* = 13) or with *Vibrio*^∆ACD^ (*N* = 12), the latter two measured at 24 hpi. For wild-type *Vibrio* compared to *Vibrio*^∆ACD^, the ratio of mean vent widths is larger by a factor of 2.4 ± 0.3 (*P* = 3.2 × 10^−5^). “x”s indicate measurements of individual zebrafish; solid symbols and error bars indicate the mean and standard error of the mean, respectively. (**C**) Representative brightfield images of the vent region, as in (**A**), highlighting the presence of rounded cells (some indicated by arrowheads) and disordered tissue. Bar: 50 µm. (**D**) Brightfield images of the gut of a larval zebrafish mono-associated with wild-type *Vibrio* at 13, 18, and 23 hpi (top to bottom). Yellow lines, offset from the vent, indicate the vent width. The dotted orange curve indicates the approximate gut midline. Bar: 50 µm. (**E and F**) Vent width (**E**) and approximate gut length along the midline (**F**) over time for six zebrafish mono-associated with wild-type *Vibrio*. Gray: data from individual fish; Orange: mean, with error bar indicating the standard deviation. (**G**) Bright field (left) and fluorescence (right) images of the vent region of a representative zebrafish mono-associated with wild-type *Vibrio*, the same fish and region as shown in (C2). Magenta (*mpeg1*:mCherry) indicates macrophages, and cyan (acridine orange) preferentially labels apoptotic cells. Bar: 100 µm. (**H**) The total fluorescence intensity of all acridine orange-positive cells, binned by anterior-posterior position relative to the vent, for zebrafish mono-associated with wild-type *Vibrio* or with *Vibrio*^∆ACD^. Solid lines indicate average values and shaded bands show the standard error of the mean for *N* = 8 and *N* = 7 fish for wild-type *Vibrio* or with *Vibrio*^∆ACD^, respectively. (**I**) The fraction of macrophages that are acridine orange-positive for zebrafish mono-associated with wild-type *Vibrio* or with *Vibrio*^∆ACD^ (*N* = 8 and *N* = 7, respectively). “x”s indicate measurements of individual zebrafish; solid symbols and error bars indicate the mean and standard error of the mean, respectively; *P* = 0.79.

Comparing at 6 dpf initially germ-free zebrafish inoculated 24 h prior with *Vibrio* or *Vibrio*^∆ACD^, or kept germ-free, we observed a pronounced enlargement of the vent (Fig. 4A), which was on average 2.5 times wider in fish colonized with *Vibrio* (Fig. 4B). In addition, *Vibrio*-inoculated fish showed numerous rounded cells in the vicinity of the vent, likely indicating cell death, not evident in *Vibrio*^∆ACD^-colonized fish (Fig. 4C). Live imaging starting at 13 hpi showed steady, progressive vent widening in *Vibrio*-inoculated fish (Fig. 4D and E). The overall length of the gut, measured along the midline from anterior to posterior, decreased over this period (Fig. 4F).

Suspecting intestinal cell death, we stained live fish with acridine orange (Materials and Methods), a membrane-permeable dye that fluoresces in acidic lysosomal vesicles, thereby preferentially staining apoptotic cells (25). Examination of the vent revealed considerable acridine orange signal in fish colonized with *Vibrio* compared to *Vibrio*^∆ACD^ (Fig. 4G and H), indicating ACD-mediated cell death.

We hypothesized that macrophages may be prevalent among the dead cells, both because of the demonstrated ability of bacteria to kill macrophages using the T6SS ACD in *in vitro* cultures (26) and because such an effect would reduce the number of macrophages and therefore, like in the ∆*irf8* fish, could explain increased gut contractions. However, examining the fraction of macrophages (*mpeg1:mCherry*^+^ cells) that showed acridine orange staining, we found no difference between fish colonized with *Vibrio* compared to *Vibrio*^∆ACD^, the mean fraction being less than 20% in either case (Fig. 4I). Even limiting the analysis to the posterior 300 µm of the gut, the fractions (mean ± s.e.m.) are nearly identical: 0.21 ± 0.05 for *Vibrio* and 0.19 ± 0.04 for *Vibrio*^∆ACD^ (*P* = 0.91). *Vibrio*-mediated cell killing via the ACD therefore does not specifically deplete macrophages.

### *Vibrio* induces redistribution of macrophages

Though simple assessment of mCherry fluorescence intensity in *mpeg1:mCherry* fish suggested that *Vibrio* induced greater macrophage numbers at the vent (Fig. 3D through F), of which a normal fraction are alive (Fig. 4I), epifluorescence imaging cannot resolve individual cells and so cannot distinguish between greater numbers and greater *mpeg1* expression. We therefore turned to light sheet fluorescence microscopy, acquiring three-dimensional, cell-resolved image stacks spanning the entire larval gut (approximately 1,500 × 400 × 400 μm^3^, or 10,000 × 2,400 × 400 pixels).

**Fig 5:**
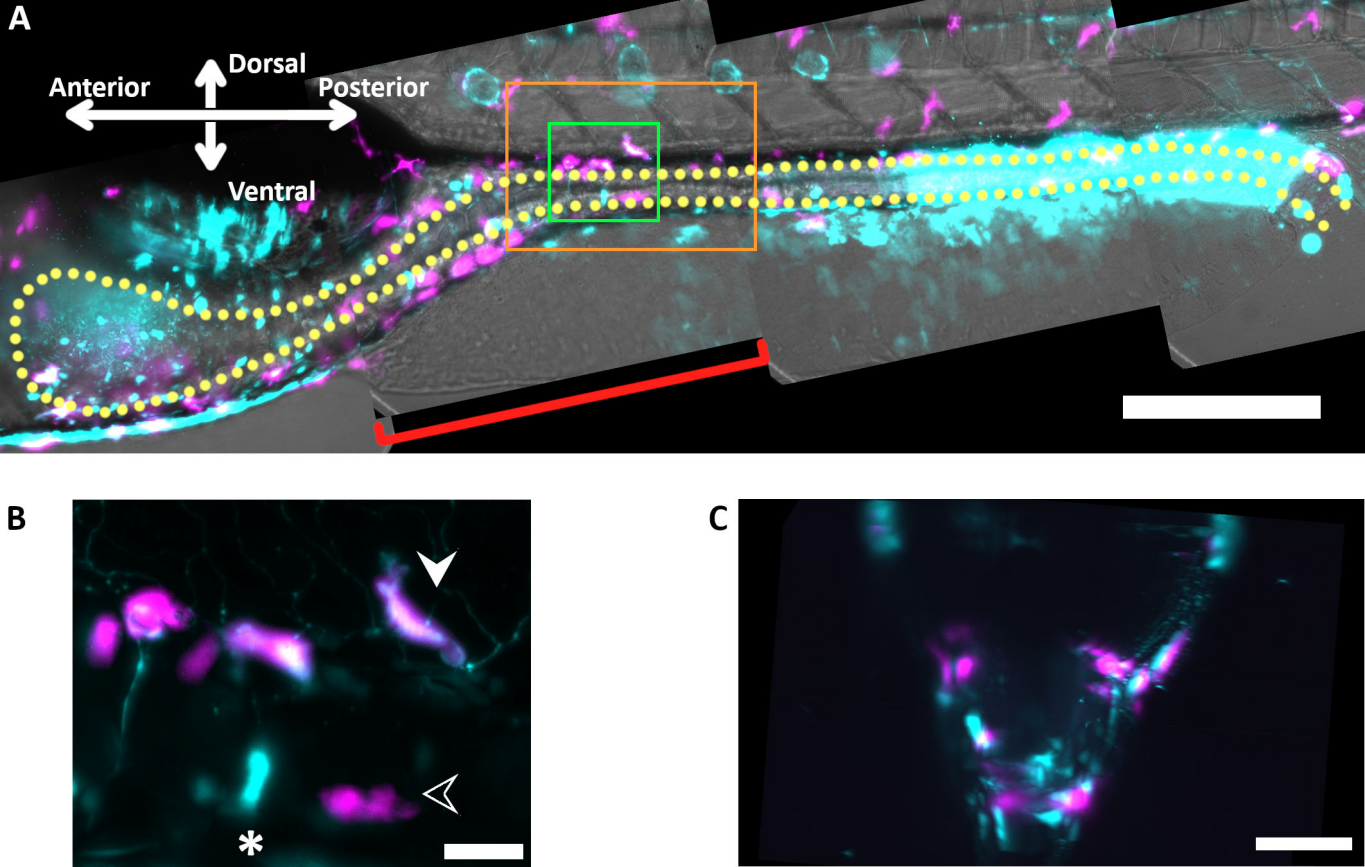
(**A**) Representative composite maximum intensity projection from light sheet fluorescence microscopy of macrophages and *tnfa* expression in a live 5 dpf larval zebrafish 9.5 h after mono-association with wild-type *Vibrio*. Magenta (*mpeg1:mCherry*) indicates macrophages, and cyan (*tnfa:GFP*) indicates *tnfa* gene expression as well as fluorescence from approximately 1% of *Vibrio* bacteria, inoculated at a ratio 1:100 GFP-labeled and unlabeled cells, only visible in this projected and down-sampled image as a diffuse cloud in the anterior. Gray is brightfield. The yellow dotted line approximately outlines the gut. The full three-dimensional image data set is stitched from four image stacks that together span the entire gut and surrounding tissue. 3D visualizations of each of the regions are provided as Supplementary Movies S5 to S8; Movie S4, of the entire second region (red bracket), is particularly useful for viewing the orientation of cells relative to the gut tube. (**B**) A maximum intensity projection along the lateral axis, as in panel **A**, of the region outlined in green in panel **A**, including *tnfa*-positive (solid arrowhead) and *tnfa*-negative (open arrowhead) macrophages, as well as a non-macrophage amoeboid *tnfa*-positive cell (asterisk). (**C**) A maximum intensity projection along the anterior-posterior axis, that is, rotated 90° relative to panel **A**, of the region outlined in orange in panel **A**, highlighting the location of macrophages and *tnfa*-positive cells around the gut.

We first provide an example that clarifies the positioning of cells relative to the gut. Figure 5 shows representative images from viewing macrophages (*mpeg1:mCherry*) and *tnfa* gene expression (*tnfa:GFP*) in live 5 dpf larvae at 9.5 h after inoculation with wild-type *Vibrio*. Rotating views of full three-dimensional image stacks are provided as Movies S5 to 8. In the example shown, 1% of the *Vibrio* inoculum consisted of GFP-expressing bacteria, whose small size and low emission intensity allow them to be easily distinguished from host cells. We note that the *tnfa* transcriptional response is complex; expression is evident in many cells other than macrophages including amoeboid cells (likely neutrophils; Fig. 5B), cells that resemble neurons, epithelial cells at the posterior end of the gut, and rosette-like mantle cells associated with the neuromasts of the lateral line. Our assessment of *tnfa*-positive macrophages considers only macrophage activation status, not other aspects of *tnfa* gene expression. A view along the gut axis (Fig. 5C) highlights the location of macrophages and *tnfa*-positive cells around the gut tube, consistent with their participation in activities triggered by intestinal microbes.

**Fig 6:**
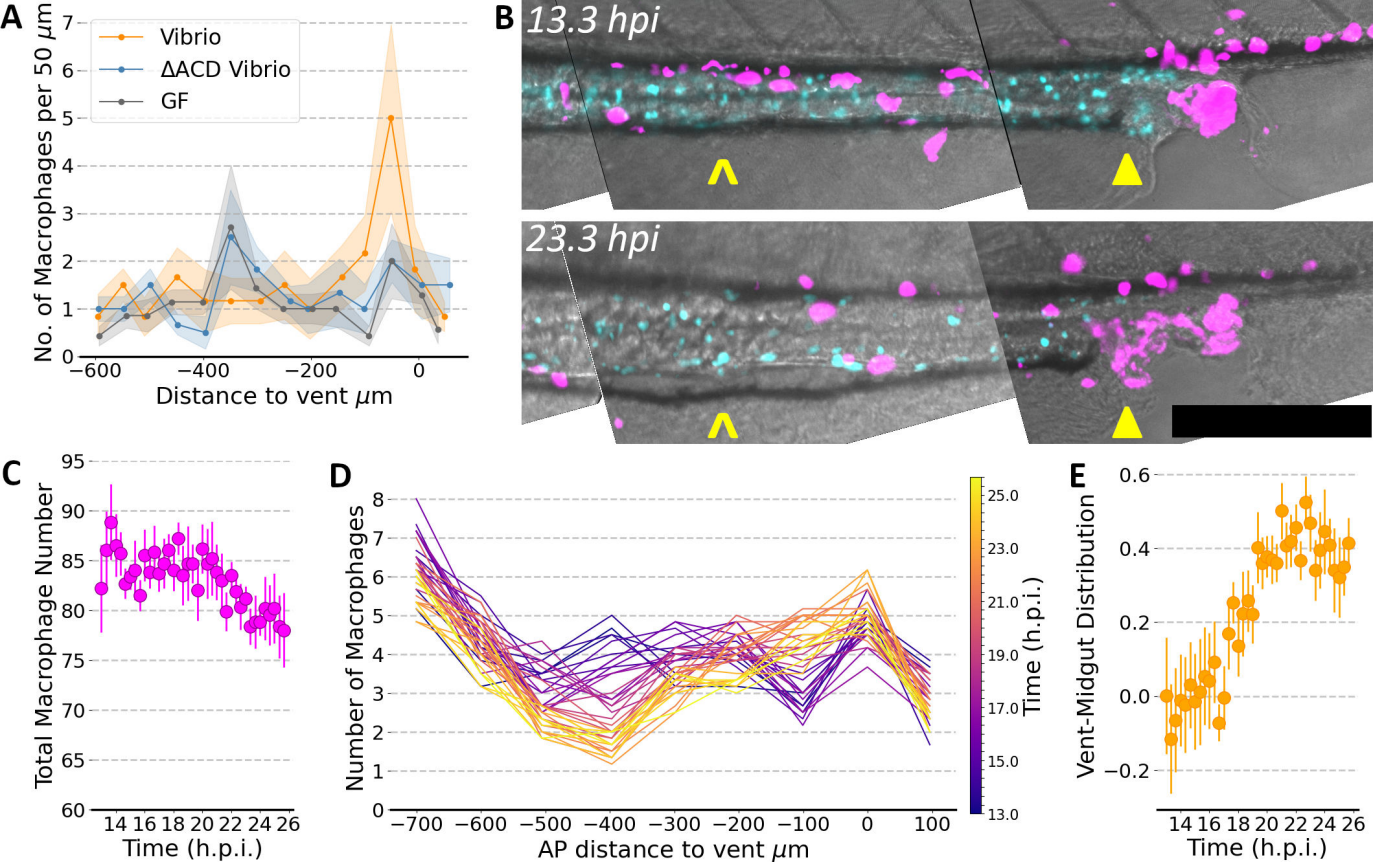
(**A**) The spatial distribution of macrophages in larval zebrafish, germ-free (GF) or mono-associated with wild-type *Vibrio* or with *Vibrio*^∆ACD^, binned by anterior-posterior position relative to the vent. Fish were fixed and imaged 24 hpi, or at the equivalent time for germ-free fish. Solid lines indicate average values and shaded bands show the standard error of the mean for *N* = 6 and *N* = 6 fish for wild-type *Vibrio* or with *Vibrio*^∆ACD^, respectively, and *N* = 7 germ-free fish. (**B**) Representative composite images from live imaging of larval zebrafish mono-associated with wild-type *Vibrio*. The images shown, maximum intensity projections of the posterior 800 µm of the gut at 13.3 hpi (upper) and 22.3 hpi (lower), are subsets of data sets that comprise three-dimensional image stacks that span the entire gut, acquired every 20 min for 13 h. Magenta (*mpeg1:mCherry*) indicates macrophages, and cyan (*phox2bb:GFP*) indicates enteric neurons; gray is brightfield. Note the depletion of macrophages in the midgut (carat symbols) and the accumulation near the vent (solid triangles). Bar: 200 µm. (**C–E**) These panels show quantification of cell numbers and positions assessed from images spanning the entire gut obtained from live imaging of *N* = 6 initially germ-free wild-type zebrafish mono-associated wild-type *Vibrio*. (**C**) The total number of macrophages over time; circles indicate the mean; bars indicate the standard error of the mean. (**D**) The spatial distribution of macrophages over time. Each curve indicates the number of macrophages, averaged over *N* = 6 fish, as a function of anterior-posterior position relative to the vent, with color indicating time from 13 to 26 hpi. (**E**) The relative distribution of macrophages as a function of time, shown as the number of macrophages in a 200 µm posterior region (−150 to +50 µm relative to the vent) minus the number in a 200 µm midgut region (−500 to −300 µm), normalized by the total number of macrophages in these regions.

To analyze *Vibrio*-induced pathology specifically in the distal intestine, we used light sheet fluorescence microscopy to image 6 dpf larval zebrafish all fixed in paraformaldehyde at 24 hpi, segmenting the resulting three-dimensional image stacks to identify each macrophage (Materials and Methods). Fish inoculated with *Vibrio*, compared to *Vibrio*^∆ACD^ or germ-free fish, showed a strong peak in macrophage density in the vicinity of the vent, over two times greater than the other conditions (Fig. 6A).

The above observations spurred us to monitor macrophage positions in live fish over the entire larval gut and vicinity over several-hour timespans. The rapid acquisition speed of light sheet fluorescence microscopy allows imaging without blurring from peristaltic motions, and its low phototoxicity enables imaging over long durations (18, 27–29). We imaged 5 dpf transgenic fish with labeled macrophages and enteric neurons [*Tg(mpeg:mCherry);Tg(phox2bb:GFP)* (30, 31)], mono-associated with *Vibrio*, from 13 to 26 hpi at intervals of 20 min. We observed accumulation of macrophages near the vent as time progressed. Strikingly, this accumulation was accompanied by a depletion of macrophages from the midgut, leaving the enteric neurons lining the gut in this region relatively devoid of nearby macrophages (Fig. 6B). Quantifying the macrophage numbers and spatial distribution verified that the total number of macrophages remained roughly constant over the imaging duration (Fig. 6C), while the midgut population steadily declined as the posterior vent population increased (Fig. 6D and E).

## DISCUSSION

Through a series of experiments based on imaging and genetic manipulation of both bacteria and host, we examined the link between a dramatic amplification of intestinal mechanical activity and a eukaryote-targeting bacterial effector, the type VI secretion system actin crosslinking domain. We observed that depletion of macrophages and the presence of the ACD each spur a roughly 100% increase in gut contraction strength that is not further enhanced by imposing both factors together. In contrast to their impact on the magnitude of contractions, macrophage depletion and the *Vibrio* ACD each leave contraction frequency unchanged. We found that *Vibrio’s* T6SS ACD induces host tissue damage and cell death while activating and not specifically killing macrophages. Rather than being selectively killed, intestinal-resident macrophages move from the midgut to the posterior site of tissue damage. These findings point to a parsimonious model: normally, macrophage proximity to enteric neurons downregulates the strength of mechanical contractions, possibly via the previously studied BMP2 pathway (21). T6SS/ACD-mediated tissue damage stimulates an innate immune response, recruiting macrophages and thereby removing the downregulatory signal.

From the perspective of the host, this sequence of events provides a simple means of coupling cellular distress to the purging of intestinal contents. From the perspective of *Vibrio*, the purging can displace bacterial competitors leaving it, being motile and planktonic, able to persist in the gut (7, 12, 16). Notably, this connection between bacterial cellular activity and neuromuscular control does not require any special biomolecular signaling pathways, instead harnessing cellular spatial reorganization to reshape intercellular communication. Of course, altered signaling may occur, especially on longer timescales. In *Drosophila melanogaster*, recent work has shown that *Vibrio* can stimulate host BMP signaling in intestinal epithelial cells via its T6SS, and that in zebrafish as well as in fruit flies the T6SS hinders epithelial cell proliferation and tissue repair (32). We also note that while this zebrafish native *Vibrio* lacks cholera toxin, it can express other virulence factors such as hemolysin, so infection by *Vibrio*^∆ACD^ should not be considered equivalent to an uninfected state.

In addition to changes in peristaltic contractions, pathogen-induced mechanical forces can manifest in other wide-ranging pathophysiologies. Our observation that *Vibrio* induces intestinal shortening, in addition to vent widening, is reminiscent of the colonic shortening that is a hallmark of inflammatory bowel disease in humans (33), evident also in mouse models where it is used as a marker of disease progression (34). Concurrent gut shortening and vent widening suggest that the gut is normally under tension, and that *Vibrio*-induced damage leads to a loss of tissue cohesion to balance pulling from the anterior/ventral directions. While peripheral to this study, testing this idea in larval zebrafish may give insights into the mechanics of intestinal disease progression.

Many questions remain regarding the spatial and temporal relationships between *Vibrio* activity and host responses. Though *Vibrio* is largely anterior-localized, it is highly motile and found throughout the gut (16), including the vicinity of the vent. Determining the timing of T6SS expression, the number of T6SS-expressing bacteria necessary to induce macrophage displacement, and the time required for the onset of increased gut mechanical activity will likely illuminate the dynamics of gut microbe and host interactions. As noted earlier, our generation of ∆*irf8* “crispants” gives zebrafish with half the normal number of macrophages. Other methods may allow tuning macrophage populations to further explore their control of intestinal motility. Macrophage numbers in homozygous *irf8* null mutants created by TALEN-mediated targeting are reduced by more than half, but not entirely depleted, at 7 dpf (22), and liposome-mediated delivery of clodronate allows the rapid killing of macrophages (35). One temporal characteristic of the host that appears to be independent of either *Vibrio* colonization or macrophage depletion is the frequency of gut contractions (Fig. 1E and 2E); we predict that these additional manipulations of macrophages would not alter contraction frequency. In contrast, we predict that manipulating the interstitial cells of Cajal, situated in smooth muscle layers surrounding the gut and believed to serve as intestinal pacemakers, would alter contraction frequency independent of macrophage inputs.

Our experiments examine a natural microbial stimulus in a living animal model, implicating dynamic alterations of neuro-immune relationships. Crosstalk between the nervous and immune systems is increasingly realized to be an important aspect of animal function (36–38). In recent years, a variety of chemokines, cytokines, and neuropeptides have been identified as mediators of this crosstalk (36). Though challenging to study, spatial and temporal signatures of inter-system interactions are likely also crucial, given the elaborate geometry of innervation and the motility and morphological plasticity of immune cells. Using intravital imaging in mice, Kulalert et al. recently showed that commensal-specific T lymphocyte sphericity, activation, and proximity to nerve fibers are all interlinked, contributing to the skin’s response to its microbiome (39). We suspect that many more spatial aspects of neuro-immune interactions, potentially modulated by host-associated microbes (5), await discovery.

## MATERIALS AND METHODS

### Animal care

All experiments with zebrafish followed standard procedures (40) and were performed in accordance with protocols approved by the University of Oregon Institutional Animal Care and Use Committee (protocol numbers AUP-22-02 and AUP-20-16). Fish from which the larvae used in these studies were derived were maintained in the University of Oregon Zebrafish Facility at 28°C with a 14-h/10-h light/dark cycle. All zebrafish in the Zebrafish Facility are offered rotifers by age 6 dpf and are offered brine shrimp (*Artemia franciscana*) in addition to rotifers from 11 dpf. Fish aged 11 dpf through 20 dpf receive brine shrimp early morning, rotifers mid-morning, brine shrimp early afternoon and rotifers late afternoon, for a total of four feedings each day. Fish aged 20 dpf through 42 dpf are fed brine shrimp two times per day. Fish from 42 dpf are offered encapsulated pellets (Zebrafish Diet 0.5 mm pellet, Zeigler, Gardners, PA) and continue to be fed brine shrimp.

Wild-type zebrafish are AB × Tu. The transgenic line *Tg(mpeg1:mCherry*), allele number: gl23, is described in reference 41. The transgenic line *Tg*(tnfα:*GFP);Tg(mpeg1:mCherry*), allele number pd1028Tg, is described in reference 42. See also descriptions of the *Tg*(tnfα:*GFP*) line in reference 43.

### Gnotobiotic techniques

Wild-type AB, *Tg(mpeg1:mCherry*), or *Tg(tnfα:*GFP)*;Tg(mpeg1:mCherry*) zebrafish were derived GF and subsequently colonized with bacterial strains, following established protocols (44). In brief, fertilized eggs were collected and incubated in sterile embryo medium (EM) containing 100 µg/mL ampicillin, 10 µg/mL gentamycin, 1 µg/mL tetracycline, 1 µg/mL chloramphenicol, and 250 ng/mL amphotericin B for approximately 6 h. Following this incubation period, embryos were thoroughly rinsed first in sterile EM containing 0.003% sodium hypochlorite and then in sterile EM containing 0.1% polyvinylpyrrolidone-iodine. These sterilized embryos were then distributed into T25 tissue culture flasks, with a density of one embryo per mL in 15 mL of sterile EM. Notably, during the experiments, the embryos relied on yolk-derived nutrients and were not provided with external feeding. The sterility of the flasks containing larval zebrafish was inspected prior to the experiments.

### Bacterial growth and zebrafish inoculation

*Vibrio* (ZWU0020, PRJNA205585) was previously isolated from the zebrafish intestinal tract. To prepare the bacterial strains for colonization at specific time points, cultures were first grown overnight in Luria Broth (LB) under agitation at a temperature of 30°C. Then, bacterial cultures were pelleted through centrifugation for a duration of 3 minat 7,000 × *g*, followed by two rounds of washing in sterile EM. An inoculum of 10^6^ colony-forming unit per milliliter (CFU/mL) was used for zebrafish inoculation, directly introduced to the flask water.

### Molecular and genetic manipulation

Unless otherwise specified, standard molecular biology techniques were employed. Reagents were used in accordance with the manufacturer’s instructions. We used restriction enzymes and various other reagents for tasks such as polymerase chain reaction (PCR) and nucleic acid modifications. The reagents were primarily obtained from New England BioLabs. To purify plasmids and PCR amplicons, we used kits from Zymo Research. DNA oligonucleotides were synthesized by Integrated DNA Technologies. The sequencing of cloned genes was verified by Sanger sequencing, conducted by Sequetech. A Leica MZ10 F fluorescence stereomicroscope, equipped with a 1× objective lens, was used for the screening of fluorescent bacterial colonies. Genome and gene sequences were retrieved from “The Integrated Microbial Genomes & Microbiome Samples” (IMG/M) website (45).

### Construction of *Vibrio* ZWU0020 T6SS mutant variants

The construction of *Vibrio* ZWU0020 mutants containing markerless deletions of either the large T6SS gene cluster or an in-frame deletion of the (ACD of VgrG-1 was accomplished using allelic exchange and the pAX2 allelic exchange vector as previously described (46). Allelic exchange cassettes targeting each locus were created using splice by overlap extension (SOE). For the large cluster deletion mutant, the following primer pairs were used to amplify 5′ and 3′ flanking homology regions: (5′ HR) 5′-AGTGAAGCAATCGGGCAGT-3′ +5′- CACACAAAAACATGACTCTGGAAATTTAATTCTGTCCTCATCGGTTAGTAAAATG-3′ and (3′ HR) 5′-CATTTTACTAACCGATGAGGACAGAATTAAATTTCCAGAGTCATGTTTTTGTGTG-3′ +5′- GGTTCGGTATAGTGGCAGCA-3′. For the ACD deletion mutant, the following primer pairs were used: (5′ HR) 5′-CCCAATGATAGCCACGGTTG-3′ +5′-CCATTCCATTTTCCACTAGGCTAAAGGACACACCTT-3′ and (3′ HR) 5′-AAGGTGTGTCCTTTAGCCTAGTGGAAAATGGAATGG-3′ +5′-GGCGCAAGATTTTCAATCA-3′. Each of the resulting 5′/3′HR amplicon pairs was spliced together by SOE and ligated into the pAX2 allelic exchange vector, resulting in pAX2-ZWU0020-T6SSlc (pTW454) and pAX2-ZWU0020-ACD..

Each pAX2 vector was introduced into *Vibrio* ZWU0020 via conjugation utilizing *Escherichia coli* SM10 as a plasmid donor strain as previously described (46). In brief, *Vibrio* and SM10 donor strain were mixed 1:1 on a filter disk and placed on tryptic soy agar (TSA). The mating mixture was then incubated at 30°C overnight after which bacteria were recovered and spread onto TSA containing gentamicin. These plates were incubated overnight at 37°C to select for *Vibrio* merodiploids. Isolated merodiploid colonies were screened for successful deletion of the large T6SS gene luster or ACD. Putative mutants were genotyped through PCR with primers flanking each locus to produce differently sized amplicons, representing wild-type and mutant alleles. Primers used for genotyping the large cluster deletion mutant were: 5′-AGTGAAGCAATCGGGCAGT-3′ +5′-GGTTCGGTATAGTGGCAGCA-3′. Primers used for genotyping the ACD deletion mutant were: 5′-CGGAGCTTTGGTCAATCTCA-3′ +5′-AGGTCTCTCCGTGGAAAACA-3′.

### Quantifying T6SS-mediated bacterial killing *in Vitro*

Overnight cultures of *Aeromonas* ZOR0001 *attTn7::sfGFP* (46) and either wild-type *Vibrio*, *Vibrio*^∆ACD^, or *Vibrio*^∆T6SS^ were mixed at a ratio of 1:3 (*Aeromonas:Vibrio*). Mixtures were washed once in 0.7% saline, serially diluted, and spotted onto TSA (5 µL/spot). Spot dilutions were incubated overnight at 30°C and imaged with a Leica MZ10F fluorescence stereomicroscope equipped with a K5 Microscope Camera.

### Culture-based quantification of intestinal bacterial populations

Dissection of larval guts was done in accordance with established procedures (47). Dissected guts were carefully harvested and transferred to 1.5 mL tubes, each containing sterile 0.7% saline solution and approximately 100 µL of 0.5 mm zirconium oxide beads. A bullet blender tissue homogenizer was used to facilitate homogenization, operated for a duration of 30 s at power level 4. Following homogenization, lysates were serially plated on LB agar plates and incubated overnight at a temperature of 30°C for enumeration of CFUs to determine gut bacterial load as previously described (7, 12).

### Measuring intestinal motility

Intestinal motility in the form of propagating contractile waves was assessed using DIC microscopy and image velocimetry, as previously described (48). DIC videos were recorded at 5 frames per second for 5 min at the distal end of the intestine, indicated in Fig. 1A. For analysis, we used open-source particle image velocimetry (PIV) software (49) to calculate a frame-to-frame velocity vector field from the image time series and then characterized the frequency and amplitudes of gut motions along the anterior-posterior (AP) axis using custom and publicly available software previously described (20) (https://github.com/rplab/Ganz-Baker-Image-Velocimetry-Analysis). We focus on the AP component of the vector field, averaging it along the dorsal-ventral direction to obtain a scalar velocity measure at each position along the gut axis and each time point. The frequency of gut contractions was identified as the location of the first peak in the temporal autocorrelation of motility, while the amplitude of contractions was calculated as the square root of the spatially averaged velocity power spectrum at this frequency, providing a quantitative measure of the magnitude of periodic gut motion.

### Light sheet fluorescence microscopy

Light sheet fluorescence microscopy was performed using a custom-built microscope described in detail elsewhere (50, 51). In brief, a galvanometer mirror rapidly scans one of two laser beams (448 and 561 nm, Coherent Sapphire, operating at 5 mW), which is then demagnified to create a thin sheet of excitation light that intersects the specimen. A 40 × 1.0 NA objective lens positioned perpendicular to this sheet captures the fluorescence emission from the optical section. The sample is then scanned along the detection axis, enabling the generation of a three-dimensional image. To image the entire extent of the intestine, which measures approximately 1,200 × 300 × 150 µm^3^, we sequentially image four sub-regions and computationally register the images after acquisition. Unless otherwise specified in the text, all exposure times are 33 ms with an excitation laser power of 5 mW. For all light sheet imaging, a 5.5 MP sCMOS camera was used. For time series imaging, scans occurred at 20-min intervals for 13-h durations.

### Sample handling and mounting for imaging experiments

Sample mounting followed previously established protocols (16, 50). Larval 6 dpf zebrafish were carefully removed from the culture flask and immersed in sterile EM containing 120 µg/mL tricaine methanesulfonate (MS-222) anesthetic. Each specimen was then briefly immersed in 0.7–1.0% low melt agarose drawn into a glass capillary, which was then mounted onto a sample holder. The agar-embedded specimens were partially extruded from the capillary to ensure that the excitation and emission optical paths did not cross glass interfaces. Larvae in the set gel were extruded from the end of the capillary and oriented such that the illumination laser sheet entered from the ventral side. The specimen holder can hold up to six samples simultaneously, all of which are immersed in sterile EM maintained at a temperature of 28°C. All long-term imaging experiments were conducted overnight, typically beginning in the late afternoon.

### Mounting for stereoscope imaging

Each anesthetized specimen was embedded in 4% methylcellulose on a microscope slide. Two stereomicroscopes were used, a Leica MZ10 F fluorescence stereomicroscope equipped with 1×, 1.6×, and 2.0× objective lenses or a Nikon SMZ25 stereomicroscope with a 1.0× objective lens.

### *Irf8* sgRNA Cas9 injections

All sgRNAs used were previously designed and characterized by Keatinge et al. (52). sgRNAs used are as follows: 5′-ATAAAGCTGAACCAGCGACATGG-3′ and 5′ TGGTGAGCAGTCCATGTCAGTGG-3′. The RNA oligonucleotides were resuspended to 20 µM in nuclease-free water and stored at −20°C until use. For *in vivo* applications, 1 nL of an injection mixture composed of 1 µL of each sgRNA, 1 µL of Cas9, 1 µL of Phenol Red (0.125%) were injected into the yolk at the one-cell stage. Phenol Red was used to screen for injected developing embryos during the GF derivation process.

### TNFα and macrophage quantification from dissected intestines

*Tg(tnfα:GFP);Tg(mpeg:mCherry)* larval zebrafish were mounted for stereoscope imaging. Their intestines were dissected and imaged using a Leica MZ10 F fluorescence stereomicroscope as described above. The number of GFP-positive, mCherry-positive, and double-positive cells were quantified visually using ImageJ.

### Image analysis

Macrophages were identified in two-dimensional fluorescence stereomicroscope images (e.g., Fig. 2A and B) using custom code written in MATLAB, available at https://github.com/rplab/Misc_code_public. First, a threshold of twice the median pixel intensity minus the minimum intensity was applied to distinguish potential cells from background. After a 2 pixel morphological opening, connected above-threshold pixels were identified as objects, and those objects with an effective radius outside the range 3–20 μm were eliminated. Separately, a high-pass filter of Gaussian width 6 µm was applied to the original image, and pixels more than five standard deviations above the median were retained and grouped into objects of connected pixels. The high pass filtering helps distinguish cells from particularly bright and broad autofluorescent intestinal background. Cells were identified as the retained objects from either of the two analysis paths, and the resulting output was visually examined to assess its reasonableness.

Macrophages and acridine orange positive cells were identified from three-dimensional light sheet fluorescence microscopy images using custom code written in Python using the open-source packages NumPy, SciPy, scikit-image, and pandas. Code is available at https://github.com/rplab/Cell_Segmentation_3D_2024. First, each 2D slice of the z-stack was down-sampled by a factor of 4 in both the *x* and *y* directions to reduce segmentation time. Next, background subtraction was performed by subtracting the median value of each 3D image from each pixel value. The images were then denoised using a local median filter. Objects were then segmented using simple thresholding with a threshold value set to the mean plus 10 times the standard deviation of the pixel intensities. To connect pseudopodia to the main cell body, a series of image operations were performed on the segmented objects, including dilation, skeletonization, and object joining, and the resulting output was visually examined to assess its reasonableness. All analysis was performed using custom code written in Python.

### Acridine Orange staining in live larval zebrafish

To visualize apoptosis in live larval zebrafish, we used the dye Acridine Orange dye (53). Larval zebrafish were derived germ-free and mono-associated with the appropriate bacterial strain. Larvae at 6 dpf were anesthetized then immersed in sterile EM containing 0.3125 µg/mL Acridine Orange for of 10 min in the dark. Following the staining, the larvae were washed in sterile EM three times to remove excess dye.

### Data and statistical analysis

Custom code written in Python was used for data analysis and plotting. All *P* values reported are from Mann–Whitney *U* tests, which are nonparametric and so do not assume normally distributed data.

## Data Availability

Numerical values of all data points plotted in Fig. 1 to 6 are in a CSV file provided as supplemental material. Raw image data can be made available by request to the corresponding author. Analysis code is available in the public Github repositories noted in Materials and Methods, specifically, https://github.com/rplab/Ganz-Baker-Image-Velocimetry-Analysis for intestinal motility analysis, https://github.com/rplab/Misc_code_public for 2D stereoscope image analysis, and https://github.com/rplab/Cell_Segmentation_3D_2024 for 3D cell identification.

## SUPPLEMENTAL MATERIAL

Data points mbio.02419-24-s0001.csv Numerical values of all data plotted in Fig. 1-6.

Supplemental Information mbio.02419-24-s0002.pdf A note about plotted data, supplemental movie captions, and Figures S1-S3.

Movie S1 mbio.02419-24-s0003.avi Gut contractions of a representative 6-dpf zebrafish inoculated with wild-type *Vibrio*

Movie S2 mbio.02419-24-s0004.avi Arrows from image velocimetry analysis are superimposed on Movie S1.

Movie S3 mbio.02419-24-s0005.avi Gut contractions of a representative 6-dpf zebrafish inoculated with *Vibrio* ACD.

Movie S4 mbio.02419-24-s0006.avi Arrows from image velocimetry analysis are superimposed on Movie S3.

Movie S5 mbio.02419-24-s0007.mp4 Composite two-channel light sheet fluorescence microscopy image showing macrophages and *tnfa* expression in a live 5-dpf larval zebrafish 9.5 hours after mono-association with wild-type *Vibrio*.

Movie S6 mbio.02419-24-s0008.mp4 Macrophages and *tnfa* expression in a live 5-dpf larval zebrafish 9, as in Movie S5.

Movie S7 mbio.02419-24-s0009.mp4 Macrophages and *tnfa* expression in a live 5-dpf larval zebrafish, as in Movie S5.

Movie S8 mbio.02419-24-s0010.mp4 Macrophages and *tnfa* expression in a live 5-dpf larval zebrafish, as in Movie S5.

ASM does not own the copyrights to Supplemental Material that may be linked to, or accessed through, an article. The authors have granted ASM a non-exclusive, world-wide license to publish the Supplemental Material files. Please contact the corresponding author directly for reuse.
