# Supplementary material for: The Vibrio type VI secretion system induces intestinal macrophage redistribution and enhanced intestinal motility
Source: mBio. 2024 Nov 22;16(1):e02419-24. doi: 10.1128/mbio.02419-24 (PMC11708011; doi:10.1128/mbio.02419-24)
Supplement: Supplemental Information — A note about plotted data, supplemental movie captions, and Figures S1-S3. [file mbio.02419-24-s0002.pdf]

Supplemental Material

Bacterial Modulation of Intestinal Motility through Macrophage Redistribution

Julia S. Ngo[1], Piyush Amitabh[2], Jonah G. Sokoloff [1,2], Calvin Trinh[3], Travis J. Wiles[3], Karen Guillemin[1,4], and Raghuveer Parthasarathy[2,*]

[1] Institute of Molecular Biology, University of Oregon, Eugene, Oregon 97403, USA

[2] Department of Physics, University of Oregon, Eugene, Oregon 97403, USA

[3] Department of Molecular Biology & Biochemistry, University of California, Irvine, California, USA

[4] Humans and the Microbiome Program, CIFAR, Toronto, Ontario M5G 1Z8, Canada

*Correspondence: raghu@uoregon.edu

Contents

- A note about plotted data
- Supplemental Movie Captions (Movies 1-8)
- Supplemental Figures (S1-S3)

A note about plotted data

Numerical values of all datapoints plotted in Figures 1-5 are in a CSV file provided as Supplemental Information.

Supplemental Movie Captions

Supplemental Movie 1: Gut contractions of a representative 6 dpf zebrafish inoculated with wild-type *Vibrio*, imaged using differential interference contrast microscopy. For size, the movie has been downsampled in space (2x) and time (5x) from the original, analyzed, image set.

Supplemental Movie 2: Arrows from image velocimetry analysis are superimposed on Supplemental Movie 1.

Supplemental Movie 3: Gut contractions of a representative 6 dpf zebrafish inoculated with *Vibrio*$^{\Delta ACD}$. For size, the movie has been downsampled in space (2x) and time (5x) from the original, analyzed, image set.

Supplemental Movie 4: Arrows from image velocimetry analysis are superimposed on Supplemental Movie 3; the scale of the arrows is the same as in Movie S2.

Supplemental Movie 5: Composite two-channel light sheet fluorescence microscopy image showing macrophages and *tnfa* expression in a live 5 dpf larval zebrafish 9.5 hours after mono-association with wild-type *Vibrio*. Magenta (*mpeg1*:*mCherry*) indicates macrophages, and cyan (*tnfa*:*GFP*) indicates *tnfa* gene expression as well as fluorescence from approximately 1% of *Vibrio* bacteria, inoculated at a ratio 1:100 GFP-labeled and unlabeled cells. The full three-dimensional image dataset comprises four image stacks that together span the entire gut and surrounding tissue. This is a 3D visualization of region 1 of 4 as enumerated from anterior to posterior.

Supplemental Movie 6: Macrophages and *tnfa* expression in a live 5 dpf larval zebrafish 9, as in Movie 5. This is a 3D visualization of region 2 of 4 as enumerated from anterior to posterior.

Supplemental Movie 7: Macrophages and *tnfa* expression in a live 5 dpf larval zebrafish, as in Movie 5. This is a 3D visualization of region 3 of 4 as enumerated from anterior to posterior. Contrast limits are set to be similar to those of Movies 5-6; GFP intensities appear saturated due to strong *tnfa* gene expression in the posterior gut, but are not saturated in the raw images.

Supplemental Movie 8: Macrophages and *tnfa* expression in a live 5 dpf larval zebrafish, as in Movie 5. This is a 3D visualization of region 4 of 4 as enumerated from anterior to posterior. Contrast limits are set to be similar to those of Movies 5-6; GFP intensities appear saturated due to strong *tnfa* gene expression in the posterior gut, but are not saturated in the raw images.

Supplemental Figures

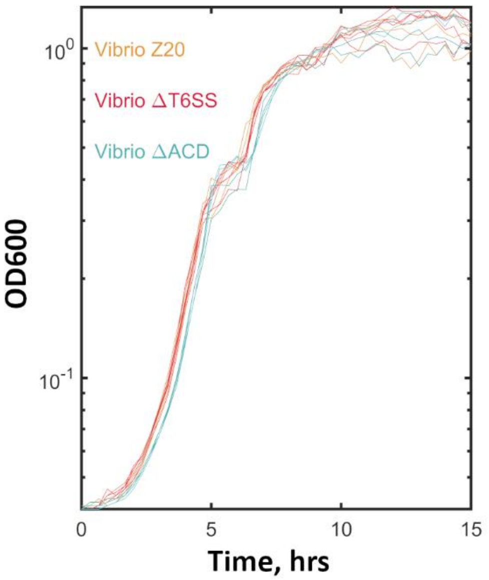

Figure S1. Growth curves, measured as optical density in lysogeny broth (LB) at 30 °C, for wild-type *Vibrio* (*Vibrio* Z20), *Vibrio* lacking the full T6SS gene cluster (*Vibrio*$^{\Delta T6SS}$), and *Vibrio* lacking the actin crosslinking domain (ACD) of the T6SS spike protein VgrG-1 (*Vibrio*$^{\Delta ACD}$), showing indistinguishable growth rates. $N = 4$ replicates were evaluated for each bacterial strain, with optical density measurements fit to logistic growth curves giving growth rates of 0.75 ± 0.02 hr.$^{-1}$ for wild-type *Vibrio*, 0.72 ± 0.03 hr.$^{-1}$ for *Vibrio*$^{\Delta T6SS}$, and 0.74 ± 0.02 hr.$^{-1}$ for *Vibrio*$^{\Delta ACD}$.

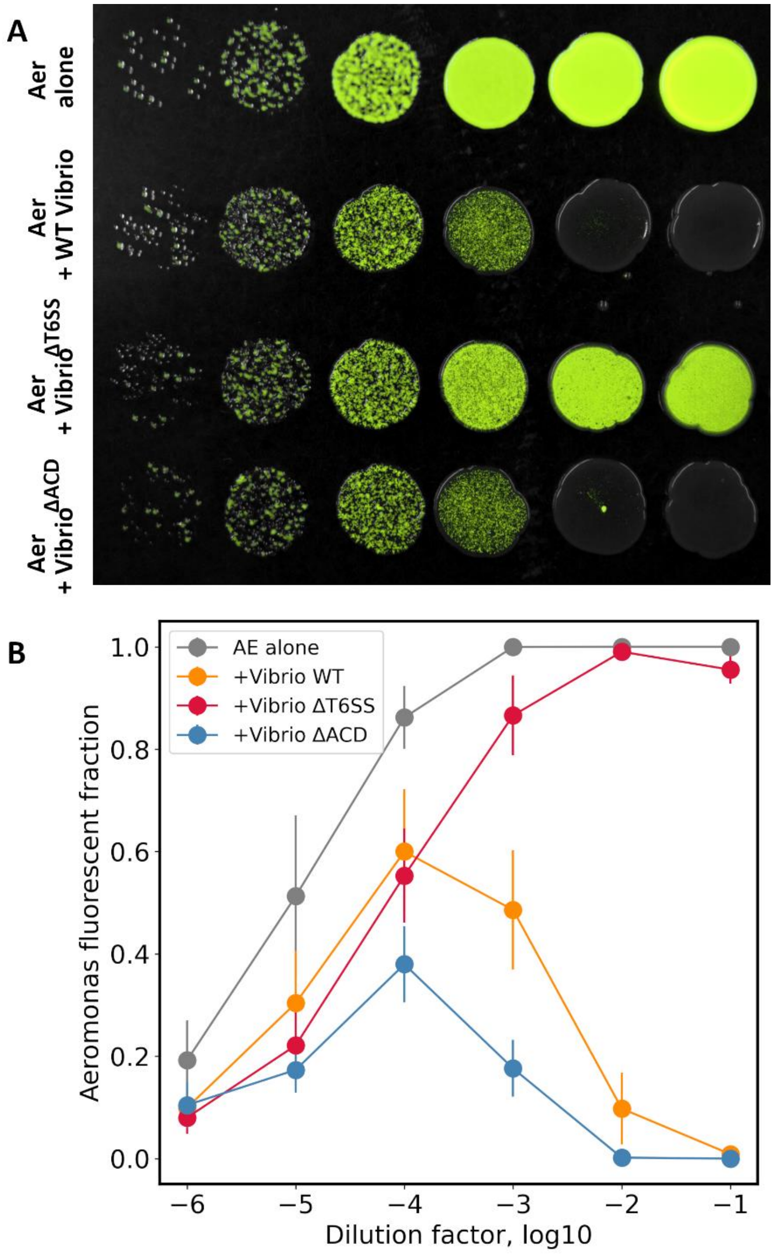

Figure S2. In vitro assays of T6SS-mediated inter-bacterial killing. (A) Images of GFP-expressing *Aeromonas* ZOR0001 spotted alone onto tryptic soy agar (top row), or together with wild-type or mutant *Vibrio* strains (lower three rows), at a series of tenfold-increasing initial bacterial concentrations (left to right). (B) Quantification of *Aeromonas* abundance as the fraction of the spotted-disk area showing GFP fluorescence. The mean (solid symbols) and standard error of the mean (error bars) across N=6 replicates are plotted as a function of dilution factor. The images and the fluorescent fractions clearly show low *Aeromonas* abundance when *Aeromonas* is co-spotted with either wild-type *Vibrio* or *Vibrio*$^{\Delta ACD}$ and high abundance with *Vibrio*$^{\Delta T6SS}$, consistent with the expectation that deletion of the actin crosslinking domain of the T6SS does not inhibit inter-bacterial killing.

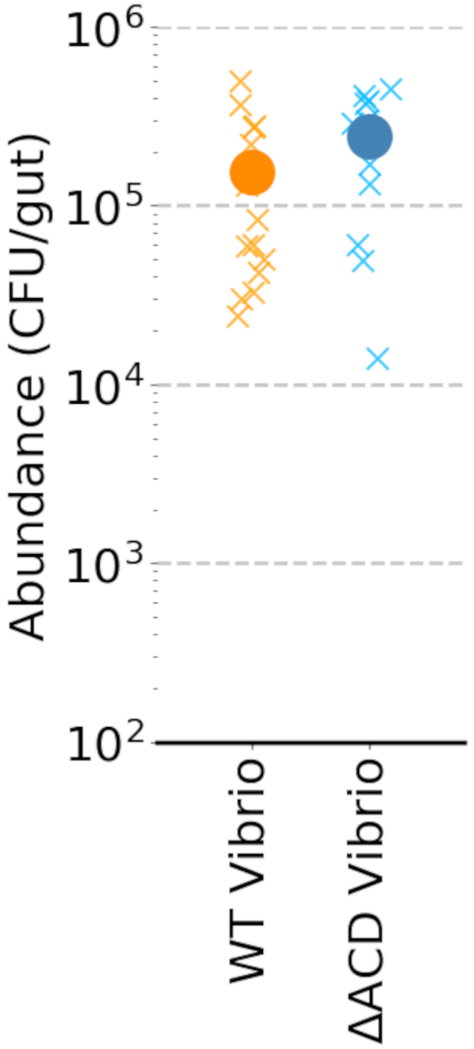

Figure S3. Bacterial abundance in the zebrafish gut for *Vibrio*$^{\Delta ACD}$ and the wild-type *Vibrio* inoculated in mono-association with initially germ-free larval zebrafish. Each "x" is derived from plating the dissected gut of a single 6 dpf zebrafish, 24 hours post-inoculation. Solid symbols and error bars indicate the mean and standard error of the mean, respectively. The mean ± standard deviation of $\log_{10}$(bacteria per gut) are 5.0 ± 0.4 and 5.2 ± 0.4 for wild-type *Vibrio* and *Vibrio*$^{\Delta ACD}$, respectively.
